# DO₂/VCO₂ Ratio Improvement on Cardiopulmonary Bypass During Minimally Invasive Mitral Valve Repair

**DOI:** 10.21470/1678-9741-2023-0464

**Published:** 2024-10-14

**Authors:** Ignazio Condello, Giuseppe Speziale

**Affiliations:** 1 Department of Cardiac Surgery, Anthea Hospital, GVM Care & Research, Bari, Italy E-mail: ignicondello@hotmail.it

**Fig. 1:**
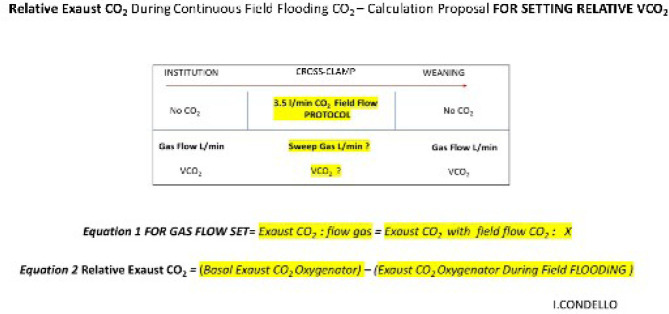
Algorithm proposal for setting relative VCO₂.

Mitral valve repair is considered the gold standard treatment for mitral regurgitation, and it correlates with a high repair rate and low mortality. The procedure can be performed per minimally invasive cardiac surgery (MICS), which demonstrates safety to treat a wide range of pathologies, including mitral valvopathy. Different studies showed that a minimally invasive approach to valve repair seems to provide equivalent earlyand long-term results to conventional median sternotomy for complex mitral valve insufficiency. In this context, in the retrospective observational study “In-Hospital Outcomes of Right Minithoracotomy *vs.* Periareolar Access for Minimally Invasive VideoAssisted Mitral Valve Repair”, by Karen Amanda Soares de Oliveira et al.^[^1^]^, including 37 patients with degenerative mitral valve regurgitation, 21 were treated with minimally invasive video-assisted approach via right minithoracotomy (RT) and 16 via periareolar access (PA); the procedures reported similar results in the two surgical techniques applied, except for the time to extubation, which was lower in patients who underwent MICS mitral valve repair via RT^[^2^]^. The presence of air microemboli in open-heart surgery during MICS correlates with the degree of postoperative neuropsychological disorder and the increase of mechanical ventilation time. The use of carbon dioxide (CO₂) in MICS is due to its high solubility and density in blood, allowing better tolerability of air embolism. The use of endocavitary aspirators during mitral valve surgery contributes to capture in the extracorporeal circuit the quantity of CO₂ continuously insufflated in the surgical field. This aspect is represented in the blood gas analysis and in the frequent correction of hypercapnia through ventilation in the oxygenator^[^3^]^. Many studies explored the association between metabolic parameters (oxygen delivery [DO₂] and carbon dioxide production [VCO₂]) during cardiopulmonary bypass (CPB) with postoperative acute kidney injury (AKI). The nadir DO₂/VCO₂ ratio < 5.3 was independently associated with AKI within a model including EuroSCORE and CPB duration^[^4^]^. In the context of MICS with continuous field flooding insufflation of CO₂, the goal-directed perfusion (GDP) for DO₂/VCO₂ becomes a challenge, in particular to establish and monitor the real parameter of the VCO₂ produced by the patient in the oxygenator due to the CO₂ administered in the field. In this letter, we introduce to scientific community an algorithm to enhance the measurement of relative exhaust CO₂ and stabilize VCO₂ using GDP and the DO₂/VCO₂ ratio during MICS for mitral valve repair (Figure 1).
